# "The Hospital" Nursing Mirror

**Published:** 1900-08-11

**Authors:** 


### "The Hospital" Nursing Mirror.

Being the Nursing Section of "The Hospital."

[Contributions for this Section of "The Hospital" should be addressed to the Editor, The Hospital, 28 & 29, Southampton Street, Strand, London, W.C., and should have the word "Nursing" plainly written in left-hand top corner of the envelope.]

### Notes on News from the Nursing World.

### THE NEW ROYAL RED CROSS NURSES.

The Queen has conferred the decoration of the Royal Red Cross upon Miss L. A. Harrison and Miss L. B. Stowell, in recognition of services rendered at Zomba and elsewhere in nursing officers and men who had taken part in the Mpeseni and Chiuta Expeditions. Miss Lizzie Bellanne Stowell was trained at Guy's Hospital, of which she was a lady pupil from June, 1894, to April, 1896, and subsequently, until November, 1897, sister. From that date she has been attached to the New Hospital, Zomba, British Central Africa.

### THE QUEEN'S JUBILEE INSTITUTE FOR NURSES.

In his capacity of chairman of the Executive Committee of the National Subscription on behalf of the Queen's Jubilee Institute for Nurses, shortly called the Queen's "Commemoration Fund," the Duke of Portland is making a special effort for this most deserving work, recently described in detail in our columns. The appeal is appropriately prefaced as follows: "'Queen Anne's Bounty' is part of our national history. It is hoped that 'Queen Victoria's nurses' may be made equally so. To secure this would not be too large a tax on our national benevolence, It would be impossible to find any work more typical of the Queen's ready sympathy and maternal interest in her people's sufferings and needs; and, lastly, it will be remembered that in making this scheme permanent we shall be handing down to those who follow after us Queen Victoria's own wish." Then, having briefly set forth the benefits of the movement to the poor in all parts of the country, and pointed out that the extension of it to every town and district in the United Kingdom is only a question of money, the committee fitly close with the letter written by Miss Florence Nightingale to the late Duke of Westminster, at that time chairman of the fund, in which she says: "Good speed to your noble effort in favour of district nurses for town and country, and in commemoration of our Queen, who cares for all. We look upon the district nurse, if she is what she should be, and if we give her the training she should have, as the great civiliser of the poor, training as well as nursing them out of ill-health into good health (health missioners), out of drink into self-control, but all without preaching, without fraternising—as friends in sympathy." Annual subscriptions, to secure a steady income, are the great need.

### THE PRINCESS OF WALES AT THE GABLES HOSPITAL.

The kindly interest of the Princess of Wales in the patients at the Gables Hospital, Surbiton, remains undiminished. On Sunday afternoon Her Royal Highness, accompanied by Princess Victoria of Wales, paid a private visit to the institution maintained by private munificence as an adjunct to the hospital ship "Princess of Wales," and were shown round the wards by Mr. and Mrs. Alfred Cooper. In the course of her visit the Princess, who had a gracious greeting for each of the 21 patients, and personally presented them with a gift on which was engraved the name of the recipient, and the battle in which he was wounded, expressed her pleasure at the improvement which she noticed in the men since she saw them at Southampton. In addition to the medical and surgical staff, the matron and nurses were introduced to Her Royal Highness.

### THE SUPPLY OF NURSES FOR SOUTH AFRICA.

Commenting upon the surprise expressed outside professional circles at the readiness with which the matron of the London produced twenty nurses for service in South Africa, the *London Hospital Gazette* says:—

"The present instance proves exactly what we have always maintained was the case. An adequate supply of suitable nurses for the war could have been obtained without any fuss if the officials had asked for the co-operation of the leading London and provincial superintendents of nursing, and left the selection of individuals in their hands. By the way, we observe that the War Office is now accepting, nay, inviting, nurses to go out whom they shortsightedly refused or ignored at the beginning of the South African troubles. Apparently we have not reached the end of the demands to be made for the 'trained female nurse' whom the War Office formerly regarded as a needless and unwelcome intruder."

### A VOLUNTEER NURSE AT MAFEKING.

An excellent idea of the nursing during the siege of Mafeking was given in our issue of July 21st by Sister Gamble, who mentioned that several ladies volunteered help. One of them, Miss Friend, has been interviewed since her return home by a representative of the *Daily News*. She states that she had three months' training at Guy's Hospital, and had taken various ambulance courses; and that, brief as her experience was, she found "even that most useful." We have not the slightest doubt that she did. A little knowledge is not necessarily dangerous, and the three months' training which a certain number of ladies undergo at Guy's, not to qualify them as nurses, but in order to obtain some rudimentary knowledge which might be useful in the event of an emergency—such as the siege of Mafeking—has frequently proved valuable.

### NURSING BY ANGLICAN SISTERS AT BLOEMFONTEIN.

Mrs. Crow Richardson, wife of Colonel J. C. Richardson, of the 3rd Glamorgan Rifle Volunteers, who soon after the outbreak of the war volunteered to go out in the capacity of a nurse, has written an interesting account of her experiences at Bloemfontein. They chiefly relate to St. Michael's, where the Anglican Sisters gave up five schoolrooms to the military authorities, which are attached to No. 10 General Hospital. Mrs. Richardson wishes to testify to "the gratitude and contentment expressed by all the men who passed out convalescent." Having affirmed that nothing is denied to the soldiers unless it is injurious to them, she makes reference to a point about which there has been some controversy. "It has been thought," she says, "that more private houses should be used for the sick during the worst emergency, but it must be remembered that there were not enough nursing sisters in the town at that time, and that two at least would have been required in each house, whereas in larger buildings one sister could supervise the orderlies and see to the comfort of about 30 patients. Besides, house to house visiting would have been almost an impossible task to the overworked medical staff, whose care and attention were unremitting."

### THE LATE MISS KINGSLEY ON SOUTH AFRICAN HOSPITALS.

An unfinished letter, written by the late Miss Mary Kingsley when she was nursing Boer prisoners, has been published. We quote a portion of it:—

"I turn to the hospital where so many deaths occurred in March and the first week in April for two main reasons—(1) The men were sent there in a moribund state; (2) the disease they were suffering from was not enteric as is commonly understood, but typhus, or camp fever, of a virulent type. There was no mistaking it—there was the characteristic rash, and it was catching, which enteric and typhoid are technically not. We have had three orderlies nearly dead of it, and several others and nursing sisters merely saved by the vigilance of the C.M.O., Dr. Carre. When I came on duty things were at their worst. With this form of fever five men died the first night, and they had been dying like flies. After a few days, however, the typhus set of patients were all dead or on the mud—mainly the latter—and I have no hesitation in saying that considering what that fever was we had an uncommon low mortality. Now we have plenty of real enteric fever cases. They are all doing extremely well, but to put down the typhus mortality as an enteric mortality and kick up a row about it, as people out here have been doing, is most absurd and unfair. It may also be possible that these enteric cases contracted the fever since they have been prisoners of war, for, as you know, at this season of the year, that fever is endemic round here, &c., but that fever that killed off the Boer prisoners was a fever they brought with them from the awful conditions they had been living under in Cronje's laager and other places up Modder River way, and, as aforesaid, they recovered far more frequently than they had any right to expect."

### EVERYTHING EXCEPT WORK.

One of the Nursing Sisters who has just arrived with sick and wounded on board the transport "Avondale Castle," says it was an uneventful passage; there was everything on board but work. The men were so far convalescent as not to require medical care, and the staff—four Nursing Sisters—waited in vain for a case. "One man had had his leg amputated, but he was hopping about quite merrily." The number (over 600) was made up of details from various regiments; indeed, hardly a regiment was unrepresented, and there were a great many Colonials.

### THE NURSES OF THE SCOTTISH NATIONAL RED CROSS HOSPITAL.

Writing from Kroonstad on June 27th, Miss Shannon, superintendent of nurses to the Scottish National Red Cross Hospital, says that so far the doctors, sisters, and orderlies are all well. They found the rapid changes of temperature very trying at first, but they are getting used to it. Miss Shannon says: "We sisters felt dreadfully cold the first night or so we slept under canvas. I couldn't sleep for the cold. Now I don't put on half so much, and I leave my tent door open. It's wonderful how soon one can accustom oneself to changes, isn't it? We being light and easily transported are here in the midst of everything, and delighted at having such chances. So after your hard work that is satisfactory, isn't it? Your heart would rejoice if you saw our nice clean beds, good bed-clothes, and the patients looking bright and clean. They say they haven't had such comfort for months. And how could they when they were on the tramp? The new sisters are all settling down to their work, enchanted with the novelty and freshness of everything. All work very happily together." There are 35 nurses, including Miss Shannon, nine of whom went from the Western Infirmary, Glasgow; ten from the Royal Infirmary, Glasgow; four from the Royal Infirmary, Perth; two from the Royal Infirmary, Dumfries; two from the Royal Alexandra Hospital, Paisley; two from the Victoria Infirmary, Glasgow; three from the Kennedy Street Fever Hospital, Glasgow; and one each from the Royal Infirmary, Edinburgh, the Royal Infirmary, Aberdeen, and St. Elizabeth's Home, Glasgow.

### A NEW HOSPITAL AT ST. VINCENT.

A sister of the Army Nursing Service Reserve, on her way to Cape Town on board the "Simla," sends us the following: "We have several Colonial sisters on board—some returning to work; whilst Sister Drury is in charge of 22 Army Nursing Reserve Sisters, about 18 of whom are going to work in South Africa. We arrived at St. Vincent early one morning, and stopped all day coaling. We were allowed on shore. Not that there is much to see, but the colouring on the hills—red and brown—is very vivid, and the more remarkable that they are quite bare of vegetation. The new hospital, to be opened in October, is well worth a visit, but the arrangements of the old hospital, which is at present in use, are very like the hospital itself—decidedly ancient. The boys here are a terrible nuisance, as they beg continually for pennies, and must be very well paid, for they can always change any number of sixpences and shillings."

### THE WAR AND THE POOR.

At a meeting of the Govan Parish Council the question of the nurses in the establishments under the direction of the council being asked whether they were prepared to join the Army Nursing Service Reserve was discussed. The House Committee had previously decided to read the letter from Lord Provost Chisholm making the inquiry to the nurses, but ultimately the decision was vetoed by the council. The great difficulty in obtaining a supply of nurses was pleaded. No doubt there was considerable force in the rejoinder of one of the council that in any case the nursing staff would know all about the letter; but we agree with the member who maintained that to formally read it to them would be tantamount to influencing them in the direction of volunteering their services. As, in the interests of the poor in the hospitals, this is not desired, the conclusion arrived at cannot reasonably be condemned.

### TO NURSE THE LEPERS.

It is announced that Mrs. Ella Clemmons has gone to Molokai with the intention of devoting her life to the tending of the lepers. For many years Mrs. Clemmons, who is a sister of Mr. Howard Gould, was an actress, but she quitted the stage when she was married. The *Globe* says that she has long given up a considerable portion of her time to missionary work, and she has now decided to exile herself entirely by throwing in her lot with the lepers. This is a great act of self-sacrifice, and Mrs. Clemmons is properly described as "a fresh disciple of Father Damien."

### THE QUESTION OF LADY VISITORS AT THE WIGAN INFIRMARY.

A gallant attempt on the part of several of the governors of the Royal Albert Edward Infirmary at Wigan to obtain the appointment of two lady visitors has not been successful for the moment. Mr. E. Walkden, who brought forward the proposal, made out a strong case in support of it. He mentioned that there are 2,000 women contributors to the infirmary, who are not at present represented at all as far as visiting is concerned, and that there are 100 male patients to whom 130 male visitors have access; while the patients in the 33 beds for women never receive a visit from one of their own sex. Not that he anticipated complaints, for he declared his conviction that it did not seem possible to have a better or a more kindly set of nurses; but he wanted the women in the wards to possess the same opportunities of complaining, if they desired, as the men already freely enjoy. Other speakers in support of the resolution also said that they did not wish to interfere with the nurses, the matron, or the doctors; but it was none the less defeated by 60 votes to 10. Mr. Walkden and his friends, however, do not intend to let the matter drop, and, if they persist in pressing it, the lady visitors will, in the end, be appointed.

### A NURSING ASSOCIATION FOR UXBRIDGE.

The people of Uxbridge have decided to form a nursing association, which will be affiliated with the Queen Victoria Jubilee Institute for Nurses. At the meeting in the Town Hall, convened for the purpose, the speakers were unanimous in testifying to the urgent need of the organisation in the interests of the poor of the district; and a strong and representative Executive Committee was elected. The question of the services of the nurses being placed at the disposal of the middle classes was raised, and Mr. P. R. Smith, one of the promoters of the movement, said that while it must be fully understood that the nurses were being provided primarily for the poor who require them, if an extreme case occurred a nurse would not be precluded from going to it. The subscriptions already promised represent a total which show that the association has not been prematurely decided upon; and, bearing in mind the size and population of the district, with the inevitable amount of illness, it is rather surprising that action has not been taken before.

### A "DISTRICT ROOM" FOR WARRINGTON NURSES.

The nurses of the Warrington District Association are to be congratulated upon the excellent premises into which they have lately moved. The new home consists of two complete houses with two communicating doors, one on the ground floor and another on the first floor. On the ground floor are the superintendent's sitting-room, the nurses' sitting-room, dining-room, tea-room, and the kitchen. Each nurse has a bedroom to herself. But perhaps the feature of the home is the "District Room," containing pegs for cloaks and bonnets, a rack for boots and slippers, rails for drying wet cloaks, cupboards for ointments, lotions, &c., for appliances, and for clothing given for the use of the patients. There is also a deep porcelain sink, fitted with hot and cold water, in which appliances that have been lent out can be cleansed and disinfected when they are returned. Ample accommodation is also provided for the indispensable bicycle.

### A "POTTOMUS IN THE NOSE."

"The other day," writes a nurse, "a woman came to the casualty ward of a London hospital. On being asked what was the matter, she said, 'Please, sir, I've had a big pottomus taken out of my nose, and I've never been right since. My nerves are just anyhow, and I'm all of a shake. I went last week to see the out-patient doctor, the gent who specials the throats and the noses. When I went into that 'ere room I never did see such a sight. The 'ead doctor hisself and a lot of young 'uns had big shiney glasses fastened with a black band round their foreheads. I says to myself, I did, 'I wonder now if these 'ere gents are a wearin' o' the Court mournin.' Well, the 'ead doctor looked at me, and then he said summat to the young gents about a pottomus, and they looked and they did their best to make me think they knew as much as he, but, bless you, they couldn't blind me; but they were nice and civil spoken. After they had all looked and talked, the doctor said, 'Missis, you have a pottomus.' 'What's that, sir,' said I? 'Anything like a hippotamus?' 'No, no,' said he. 'A puttomus is a growth, and you must have it removed.' I give my consent, but never again will I part with a bit of my flesh and blood so easily, for I've been real queer ever since—a kind of lonely like ——. Now I just fancy a drop of strengthening medicine will pull me together, please God; and He will bless you, too, sir, for listenin' so patient to a poor woman's trouble. 'Thank you, sir.'"

### SHORT ITEMS.

The death is announced of Mother Patrick, the Superior of the Dominican Sisterhood, at Salisbury, Mashonaland, who rendered great services in nursing the sick and wounded at the time of the Matabele war and rebellion.—The gift of chocolate, stamps, and writing-paper for the hospital at Springfontein, "in gratitude for faithful nursing of Lance-Corporal C. Grimes, who died at Orange River," is acknowledged in the daily papers by "Sister Barbara."—The funeral of Nurse Sage, of the Welsh Hospital, who died from dysentery, took place at Springfontein on June 14th, and was attended by a large company. The service was most impressive.—Miss E. G. Scaife, who was recently appointed charge nurse at Hunslet Union Workhouse Infirmary, writes to state that she was trained at Crumpsall Infirmary, Manchester, before proceeding to the York Home for Nurses.—At the meeting of the committee of the Pitlochry District Sick Nursing Association the other day, plans were submitted in connection with the proposed home which is to be erected as a memorial to the late Dr. W. S. Irvine, and it was agreed to invite estimates for the work.—Miss Mary Henderson, of the Army Nursing Service Reserve, who sailed for South Africa on Saturday, was trained at the Royal Infirmary, Perth, where she was afterwards sister-in-charge of the enteric and diphtheria wards. She was then charge nurse at the Brook Hospital, Shooter's Hill, for three years. At the end of that time she was engaged in private nursing in the Riviera; and on joining the A.N.S.R., was sent to the Herbert Hospital, Woolwich.

### Lectures on Medicine to Nurses.

By H. A. Latimer, M.D. (Dunelm), M.R.C.S. Eng., L.S.A.Lond.; Consulting Surgeon, Swansea Hospital; Past President of the South Wales and Monmouthshire Branch of Brit. Med. Assoc.; Past President of the Swansea Medical Society; Hon. Life Member of the St. John Ambulance Association, &c.

### THE SPECIFIC FEVERS.—THE ENTRY OF INFECTIOUS AGENTS INTO THE BLOOD.—MODES OF VISITATION OF THESE DISEASES.

It is well known to you that the air which is around us is charged with dust. I take it that dust consists of innumerable varieties of organic bodies given off from human, animal, and vegetable life, as well as of minutely formed particles of inorganic matter. With every act of breathing we carry into the respiratory passages air which is loaded with dust, and so impalpable is this matter that it is probably carried down to the finest lobules of the lungs and right into the tiny air cells, which are the chambers in which the essential acts of respiration in the exchange of pure air for used up air are effected. These air cells, or vesicles as they are called, have walls of the finest texture, which serve as supports for capillary blood vessels, and it is in these vessels that venous blood, charged with carbonic acid gas derived from blood which has made the circuit of the body and has therefore collected this impurity, becomes changed to arterial blood by the substitution of oxygen brought from the fresh air, outside the body, at each act of inspiration. Many instances are afforded us in every-day life of the facility with which vapours arriving at the lungs with the ingoing breath gain speedy access to the blood, and, through that fluid, rapidly affect the system at large. I will quote two as illustrations. Who has not experienced the lassitude, drowsiness, and headache caused by breathing the air of a crowded assembly hall? Who has not felt that something more than the preacher's voice has had to do with the sleepy state one has fallen into in a warm and crowded church? In such cases some little time has elapsed, as a rule, before the noxious effects of the vitiated air have been felt by you. But in the induction of anæsthesia by nitrous oxide gas, ether, chloroform, and other like agents, we see a rapid influence over the nervous system through the transference of vapours into the blood through the breathing organs, quickly brought about. That the air which has reached the lungs and air-cells is charged with dust is proved by the fact that when these organs are examined in the post-mortem room they are often found to be discoloured black; in coal miners this is so markedly the case that the name of anthracosis is given to the condition, and we speak of the coal miners' lung. And if I have demonstrated to you how easily the fairly large particles of coal or soot will find their way to the lung, you require little imagination to realise how readily the microscopically small organisms which constitute infective agents of disease, which are floating everywhere in the air, will find their way there, and be absorbed into the blood through the excessively delicate basement membranes of the air-cells and the very walls of the blood vessels which are distributed on the same.

Another channel of infection is through the stomach and bowels, or through the walls of the tubes leading to the same. The absorbent vessels which are to be found everywhere in the body will readily absorb any foreign bodies which lodge in their vicinity, and, having absorbed them, will convey them into the general current of the blood. The bacteria which possess the power of exciting these surgical septic fevers afford us abundant proofs of this fact. If any one fact has been more clearly proved than another in the realm of medicine and surgery it is the one that septicæmia, pyæmia, and other such affections, are due to the absorption at wound surfaces of germs which have gained access to the blood and have developed and have distributed their toxins, or poisonous substances, there. You will read that such-and-such is spread by fomites. This is the plural of a word derived from the Latin one *foveo*, to keep warm; and is defined as meaning "any porous substance capable of absorbing contagious effluvia, as goods or clothing." So when a disease is said to be spread by fomites, it means that micro-organisms which have escaped from pre-existing cases of a like kind, and have attached themselves to clothes, carpets, curtains, books, or other vehicles of infection, have become disengaged from them and have reached a new subject to carry out a fresh *rôle* of infection.

Another question I set myself to answer was as to the channels from which contagia leave the system. Here no general answer can be given, for although in the majority of the special fevers infections leave the body through the breathing, cutaneous, urinary, mammary, and alimentary tracts, there are some in which one route is chosen exclusively, and there are others where the infection has a predilection for one or more avenues, although it does not entirely adhere to it or them. Enteric fever, for instance, is infective only through the stools voided by the sufferer, and if these are carefully destroyed, and any fæcal matter adhering to his body or to the clothes or bedding is disinfected and got rid of, those who are brought into contact with him as nurses or visitors will not contract the disease. In the same way cholera spreads. On the other hand, mumps is extremely infectious through the breath, and although all discharging parts from a scarlet fever patient are infective, the desquamated or shedded skin which comes away from him is peculiarly virulent, and preserves its infective power for an unknown period of time.

There are yet other questions concerning these diseases which are of a highly interesting character. It is a curious thing that some of them have a trick of entirely disappearing, and of suddenly reappearing in our midst, whilst all of them are prone to occur as isolated cases, and to suddenly settle upon us in the epidemic form. So we speak of a disease as showing itself in a sporadic or an epidemic form, the former word being derived from the Greek "sporadikos," and meaning "something occurring singly, or apart from other things of the same kind," the latter from the Greek "epi," upon, and "demos," the people, and signifying "a disease which, arising from a widespread cause, affects many persons at once."

Of the specific fevers which completely disappear and then suddenly break out as an epidemic, measles, mumps, and whooping cough serve as good examples. In a community which has been quite free from any appearance of these affections, many cases of the one which is to appear in epidemic form will suddenly show themselves. It is obvious that the specific organisms of infection must have been lying dormant during a time of inactivity, and that they must have suddenly been rendered active by some unknown atmospheric cause. Why they should be impotent for awhile and suddenly assume virulence is more than anyone can tell in our present state of knowledge, and why at one time an epidemic visitation of one of these specific diseases will be malignant in character whilst at another it will be of a mild type is another problem which has been unsolved. Everyone who has had much experience in attending the sick, however, is aware of the fact that this difference in different visitations of these fevers does occur. I have seen epidemics of scarlet fever, for instance, in which such malignancy was displayed that every case I attended was accompanied by grave and alarming symptoms; and I have seen such an epidemic followed some months later on by another visitation in which no single invalid gave me one moment's anxiety.

### Invalided from Bloemfontein.

By a Member of the Army Nursing Service Reserve.

Since it has been my unfortunate lot to be invalided home from South Africa I should like to tell you some of my experiences. I was in Bloemfontein for five months, where one really learned from personal experience what the horrors of war are. All of us came in for a share of toil, hardships, and, in most cases, illness, but whatever we may have suffered we count as nothing, because of the splendid examples of cheery fortitude on the part of our brave soldiers—officers and men alike. Those who had most to bear and to suffer showed up best.

Nursing Under Great Difficulties.

Our nursing was certainly done under great difficulties, and often when tired and depressed by the inability of so few nurses to do all that we felt ought to be done for our patients, we allowed ourselves to grumble and be exacting. The supply of utensils required to efficiently and conveniently nurse such enormous numbers of bad enteric and dysentery cases was very great. Our chief worry and vexation of spirit was largely caused by our hospital being equipped for five hundred patients, whereas we had one thousand seven hundred men to nurse; with naturally an infinitely limited supply of the barest necessities of hospital furniture.

At Wynberg.

Having fallen sick I most reluctantly had to submit to being invalided home on my partial recovery. I tried to take this order graciously, but it was certainly not a happy moment for me. We were first sent down to Wynberg, on leaving our own hospitals, to recruit. There the arrangements, both for "Tommies" and "Sisters," whether invalids or workers, were excellent. The equipment was sufficient, and the diets were liberal.

On the "Nubia."

Three other sick sisters came home in the "Nubia" with me. We enjoyed every day of the voyage, which was as perfectly lovely as it was possible to be. No startling adventures or hair-breadth escapes from threatened dangers came in our way. Nevertheless, our luxurious daily life on board, and the glad feeling of returning to normal health proved to be joy enough to us. Of course, we welcomed the diversion of a passing ship, and the first sight of land, and talked much of the landing at Las Palmas, where we coaled and took in fresh supplies of fruit and vegetables. Then, too, mildly bargaining with the Portuguese salesmen, who came on board with their drawn-thread work, silk shawls, parrots, monkeys, &c., was exciting. Having our weights taken every few days by the butcher tying us up in a sheet like the baby in the advertisement for Mellin's Food, was always a sort of stock joke. Flying fish, spouting whales, and brightly-coloured jelly-fish all gave us pleasure, and broke the stillness which fell upon us as convalescing passengers.

An Alarm of Fire.

We were very much enlivened one day at 4.30 p.m., when the fire alarm bell was rung. The hose was brought out in a moment, everyone of the crew was in his place, doctors and sisters were at their posts in the wards of the ship, and the convalescent Tommies were ordered up on deck ready to be taken off in boats. The rest of us, who were of no use and had no place, were ordered to stay in the dining-saloon. I really thought with all this method there was some truth in the alarm, and I am proud to say I did not disgrace my profession by showing signs of excitement, or apprehension, the more so as I found afterwards that it was unexpected drill. There was an excellent library on board, and everybody seemed greedy to read every book they had time to run through. The books were varied enough to suit every style of reader one would have thought, though one officer, who grumbled at most things, complained that the library contained no Bible and no book of poems. The dining-saloon produced an abundant supply of Bibles and Prayer-books on Sundays, where service was conducted.

Burying Four Tommies.

I am afraid that poor Tommy had no library at his disposal, but as he had an excellent supply of tobacco given him through the Red Cross Society every week, he was quite happy and contented. We were unfortunate in burying four Tommies and a black boy at sea. Funerals at sea are sorry sights indeed. The ceremony of the black boy's funeral was conducted by one of his own native official superiors, who was also one of the ship's servants, as their rites are weird, long, and secretive. Naturally, as they tried to go through the ceremony without anyone being able to see what they were doing, we had our inquisitive faculties aroused, and tried to peep over the blankets which some of their numbers held up to shut out the view. We were disappointed in seeing very little.

The Evening Concerts.

"Tommy" very much enjoyed his evening concerts, which he gave two or three times a week after the first week at sea. The deck piano was taken aft, and he sang us thrilling love songs and recited poems of brave deeds done long ago. "Tommy" is without doubt a most sentimental individual, judging from the tone of his favourite songs. Perhaps as he neared home he gave a thought or two to "the girl he left behind him." The wards in the "Nubia" are most comfortable and convenient. There were about three hundred patients on board in hospital, but many of these looked very poor imitations of invalids. We came into Southampton Water at half-past seven p.m., and the delight at the view of the Isle of Wight in the calm beauty of the summer evening was very real.

### Dr. Steevens' Hospital, Dublin.

On Friday the prize awarded as the result of the examination on surgical nursing was presented to the successful probationer by Mr. W. S. Haughton, M.B., B.C.H. Miss Kathleen Hemsworth won the prize, which consisted of a beautiful wallet, fully furnished, with silver-mounted plate, name and date engraved. Marks 30 2-8, out of a possible 32. Some of the questions were: "What is meant by physiological rest?—How is it procured? How would you nurse a case of concussion of the brain? What is the use of a surgical dressing?—How would you prepare one? What are the signs of inflammation, and what is the result of inflammation of a wound? How would you prepare a patient for operation, say, abdominal section? Where would you expect to find disease germs?" Twenty-three probationers were examined, and twenty passed.

### Minor Appointments.

Dumfries and Galloway Royal Infirmary.—Miss Lucy Wardlaw has been appointed Staff Nurse. She was trained at the Sheffield Royal Infirmary, and has since been staff nurse in the Accident Hospital, Mansfield.

Walsall and District Hospital.—Miss Janet Bray has been appointed Night Superintendent. She was trained at the Royal Hospital, Sheffield, where she has since been sister in charge of the male medical ward.

Blaby and Wigston Magna Joint Hospital.—Miss B. J. Hewer has been appointed Assistant Nurse. She is just completing her training at Leigh Sanatorium.

### Last Duties to Patients.

By a Matron.

Several correspondents having asked for information and instructions respecting dressing and laying out the dead in private houses, a matron of experience has, at our request, written the following article on the subject:—

In the play the curtain falls as the hero performs his final act in the great drama; in the story, the chapter or book closes with his death; and in our hospitals, as one by one our worn-out brothers and sisters, "unwilling or willing, sure-footed or sore, surely come to the beautiful River of Rest," the screens are drawn round the bed, the weeping friends are gently led out of the ward by a kind hand, and for them, too, the curtain falls on the life that has just sighed itself out after its buffeting with the waves of this troublesome world. They go back to their desolate homes knowing that they can do no more for their loved one, and they leave perforce to other hands the performance of those last tenderly sad duties that are part of the grand work of the nursing profession.

### In the Hospital.

For the nurse's work is not done when the busy house surgeon has listened in vain for another heart beat through his stethoscope, and has gone away with a shake of his head and a sigh of disappointment at losing his case. For her the curtain has not fallen as she puts away the hypodermic syringe that has been trying to pulse back the ebbing life through the sluggish veins by means of strychnine and ether. She, too, sighs at the victory the great enemy has wrested from her striving hands; but she has no time to spare for more than a sigh. The needs of the living are ever pressing on her, so in a quick, methodical manner she performs all the last duties to the body so familiar to all hospital nurses, and in as short a time as is consistent with decency it is removed to the mortuary.

A new probationer soon gets over the first shock of performing these last offices for her patients after she has assisted at them a few times; and though it is to be hoped she will never grow callous over the work, she certainly has no time to spend in consulting her feelings. It is, in hospital, all a part of the daily routine, and has to be faced and gone through like other details of nursing.

### In Private Houses.

But when it comes to doing the same things in private houses she finds it somewhat different. Here the feelings of the patient's relatives have to be largely considered, and when, as is often the case, the nurse finds herself in a disorganised household of people distracted with grief over their bereavement, she will cause herself and her profession to fall into eternal disrepute with them should she show what has unhappily been termed "the hospital manner." I have met one or two nurses with this manner, and have found it unsympathetic, curt, and greatly to be deprecated and avoided. Then, if at any time, is the opportunity for the exercise of all womanly tact and sympathy. Do not hurry the friends out of the room as soon as death has occurred. Leave them alone for a few minutes, whilst you unobtrusively busy yourself about other things. They will soon be glad to allow you to do your part, but never seem in a hurry to begin. A few minutes more or less will make no difference to you, and a great deal to them. There will generally be someone—a servant, a housekeeper, a neighbour, or some more composed member of the family than the rest—who will volunteer to give you the assistance you require, and as soon as the rest have dropped one by one out of the room you can close the door and commence your work.

### How to Proceed.

Take the hot-water bottles, if there are any, out of the bed, remove the blankets and quilt and the night-dress, leaving only a sheet over the body. If you have been nursing the patient on a feather bed, have it taken off now, and leave the body with a piece of macintosh under it large enough to reach from the waist to the knees, lying on the mattress, the head supported by one pillow. With a little help it is not difficult to get even a large feather bed off the mattress Turn the body over on its side and roll up half the bed towards the centre, as though changing the under sheet of a patient unable to get up. Then turn the body on to its other side, and the bed can easily be slipped off on to the floor. You will probably have some lint, tow, or absorbent wool, and bandages with you, but if not, you must manage without, and make old linen or handkerchiefs serve your purpose. Cut two small pieces of lint or wool, dip them in water, close the eyes with them, and leave them there for the present. A roller bandage under the jaw, fixed so that it rests in the hollow of the throat, is a better way of closing the mouth than tying up the face; but if you have no bandage, tear a piece of old linen into an ordinary four-tailed bandage, with a hole in the centre for the chin to rest in, and tie the tails at the top of the head. Should there be dressings of any kind on the body, take them off, and apply instead to the wound a piece of absorbent wool, lightly kept in place with a bandage. If it be a tracheotomy case remove the tube and put a stitch or two in the wound to draw the edges together. Take out a Keith's or any other tube from the abdomen. Straighten the body and limbs, tying a bit of broad bandage or a handkerchief round the legs at the ankles and another just above the knees.

### Consideration for the Friends.

Do not take any rings from the fingers or ears without first asking the friends' permission to do so, and leave the body as it is for about an hour, covered only by a sheet, while you tidy the room and get ready all you require. By the end of that time the jaw will have set, and the bandages, &c., can be removed. You will now want soap and flannel, warm water, with a little disinfectant such as 1 in 20 carbolic lotion or Condy's fluid added to it, one or two towels, brush and comb, tow or cotton-wool, and a white night-dress and white stockings. Wash the body all over; put on the night-dress and stockings after plugging carefully with wool or tow such orifices as the rectum and vagina. Brush and comb the hair, arranging it as nearly as possible in the manner that the patient used to wear it when alive. Inquire if the friends wish to keep some, and if so, cut off one or two locks from the back of the head, if possible, where their absence will not be noticed. If the patient is a child or a woman you may tie the nightdress at the wrist, waist, and above the ankles with white ribbon. Lay some white flowers, if you can get a few, on the breast, and do it all with reverence and decency as you would like it done for your nearest and dearest, or for yourself.

### Respect the Religion.

When you have finished, clear everything away yourself without troubling anyone unnecessarily, and leave the room perfectly neat and tidy. Respect the religion, whatever it may be, of the patient's friends, and should they be Jews, make quite sure that they wish you to perform these last offices for the body before you begin, as they may prefer one of their own "watchers" to do it instead. Should they be particular about covering mirrors, keeping candles burning,

&c., in the room where the body is, do not thoughtlessly run counter to their prejudices. If the case has been an infectious one be doubly careful that all the body and bed-linen, &c., sent to be washed is thoroughly soaked in whatever disinfectant the doctor may have been using during the progress of the case. Pour disinfectant over it yourself in any case, leave an open dish of it in the room, and sprinkle the sheet covering the body with it. When you have put everything in order, ask the friends if they would not like to come into the room again, which they are generally anxious to do, and then you can take the opportunity to slip away and make your preparations for leaving.

### Avoid Undue Haste.

Do not, however, show undue haste to be gone, and if you see you really can be of further service to the family by staying a few hours longer, or even for a day or two, then stay; but, as a general rule, it is best to leave shortly after you have finished your last duties to the patient, for the majority of people prefer to be left alone in their grief without the presence of a stranger, however sympathetic, among them. It is not usual for a nurse to remain for the funeral, unless by very special request.

So for you, too, the chapter of that case is at last closed, and though it is disheartening to know your patient has died in spite of all your efforts to pull him through, you will ever have the satisfaction of knowing what true help and comfort your skilled nursing has been, both to the loved one gone and the friends remaining.

To be this true help to the sick and sorrowful is one of the sweetest rewards of a work that is full of much weariness and discouragement, and the remembrance of it will surely be gladness to us when it comes to the turn of each to go out alone into the Great Beyond.

---

### Convalescent Soldiers on Land and Sea.

Notes by an Alexandra Nurse.

Our correspondent who, it will be remembered, usually works at Maritzburg, but who is at present on sick leave at Durban, writes: At a time when so much is being printed in the public newspapers regarding the mismanagement, &c., of military hospitals in South Africa, it may not be uninteresting to your numerous readers to hear the general opinion of the men themselves. I have had the pleasure of visiting several of the transport ships for convalescents, and in converse with the men have only heard expressions of praise and gratitude for all that was done for them. On board the "Britannic," which sailed two weeks ago for England, I found 300 men from Maritzburg, many of them my former patients of the inspection room. The "Nubia," sailing three days later, had also many of our old friends on board. Smiles and "Sister, don't you remember me?" greeted me everywhere, followed by reminiscences of the wounds I had dressed for them, and all that they had undergone since last we met, but not a grumbling or a discontented word from any. They were rather disappointed when told I had only come on board as a visitor and was not to accompany them home, as they knew I had a very soft place in my heart for all Tommies, sick or well. I had to inspect and admire the gift of warm clothing provided for the voyage, also the chairs, lounges, &c., for the weary ones. The people of Natal have been most generous in providing comforts.

### The "Orcana."

In visiting the "Orcana" we felt quite at home. Miss Macdonald, sister in charge, and a fellow-country-woman, has a very charming manner, and seemed to have both the love and respect of all the nurses under her. After seeing over the ship, and talking to many old patients, we were introduced to the medical officer in charge, who kindly consented to our giving a concert on board the following day. This arrangement was most successfully carried out. Though a little rain fell it in no way interfered with the comfort or happiness of the convalescents, who thoroughly appreciated the various items on the programme. On Saturday, the 7th, the ss. "Greek" sailed with its living freight of convalescents. Quite a number of friends gathered on the wharf to bid them farewell. Among the friends were six of the A.N.R.S., who made quite a bright group with their scarlet-lined hoods. These had just arrived on the "Briton."

### A Patient from Ladysmith.

On the occasion of a second visit to the "Orcana" I found a patient whose wife and little daughter I had had under my care previous to their being sent home. This sergeant had been at Eschowe prior to being sent on to Ladysmith, where he was in the siege and where he had contracted very acute and painful rheumatism. He had been in hospital 113 days, and when I spoke to him he was still very lame, but able to hobble about with the aid of two sticks. I had heard so much of the absent Dada when in attendance on his wife and child that it was a pleasure to meet him now in this accidental way. On Sabbath afternoon the children of the Presbyterian Sunday-school brought a very handsome contribution of medical comforts, warm underwear, fruit, eggs, and other delicacies, which were distributed to the various hospital ships. They all looked so eager and happy that I am sure Tommy would have had a lump in his throat had he seen his young friends. A convalescent home has also been in operation here for some time past. Sister Eveline, in charge, leads a very busy life among her 13 patients, who greatly appreciate her efforts for their comfort and restoration to health.

### The Pinetown Hospital.

To-day (July 11th) I have just returned from a visit to the hospital at Pinetown, given by Mr. Moseley. Situated on rising ground above the railway line, it has a magnificent view of the adjoining country and also a peep of the sea at Durban. The patients enjoy the benefit of the sea breezes while not being too close to the sea, and the improvement in their health is very marked. A Scotch nurse, trained in the Glasgow Royal Infirmary (I also am a Glasgowegian), showed us round. Old patients greeted me here, and were loud in their praise of their surroundings and the care and attention bestowed upon them. The generous donor of the hospital has spared no expense to make the men comfortable, and their food is on a most liberal scale.

### Marriage of a Sister.

In closing I must refer to a very pleasing ceremony which took place in Maritzburg this week. A civilian sister, who has been nursing in the Camp Fort Napier for several months, was united in marriage to Dr. Strange. As befitting a military ceremony, not only the bride, but also many of the guests, were arrayed in khaki, enlivened by scarlet trimmings.

### The St. Andrew's Home Club for Nurses.

By a Special Correspondent.

As St. Andrew's Home is now rapidly approaching completion I asked Miss Edith Debenham, the founder and proprietor, to allow me to inspect the handsome and commodious building in Mortimer Street, erected under the auspices of the architect, Mr. A. J. Hopkins, in order that the readers of the "Nursing Mirror" might have the earliest possible information about it. She courteously at once consented to show me all she could, though she warned me that several of the rooms were still in a chaotic condition.

"I suppose, then, you are not yet quite prepared to receive inmates?"

Miss Debenham laughed. "No, I am not; but, as a matter of fact, several of the rooms are already full. I could not keep the occupants out. As soon as they found matters were sufficiently advanced to allow of beds being erected, they maintained that they did not mind passages, dining-room, &c., being still in the hands of workpeople, and asked me to have them at once. Many of these have, of course, lived with me before in St. Andrew's Chambers. The formal opening will be early in October."

### The Accommodation.

"How many will you ultimately be able to accommodate?"

"There are thirty-six bedrooms; four of these have two beds in each."

"Is the latter arrangement for purposes of economy?"

"No; the charge is almost the same, as the double rooms are in most cases large ones and in a good position, but sometimes friends, or sisters, like to be together, and I am often asked for a double room."

"Do you receive no one but nurses?"

"Nurses have the preference, but I have no objection to taking a limited number of other ladies, more especially if they are friends of those who are established inmates. Just now I have a number of medical students who have not left town, and to whom I have given a sitting-room of their own for the time."

### The Charges.

"Now may I hear about the charges?"

"A private sitting-room costs 3s. 6d. a day, a bed-sitting-room the same price, whilst a bedroom and sitting-room *en suite* is 7s. Single bed-rooms are 2s. 6d. and 3s. a day; double bedrooms 4s. and 5s. There is no charge for attendance and lighting, which is everywhere by electricity."

"Then I believe you let some rooms by the year?"

"Furnished rooms are from £35 a year, unfurnished ones from £24. The latter I find in great demand. I have only six, and all are let, one lady having put down her name for the next vacancy."

"The entrance fee to the club is a guinea, I see. What is the annual subscription?"

"A guinea a year, which includes use of a dining-room, with small tables, a drawing-room, a reading-room, and a morning-room, which is set apart for the use of residents and visitors staying in the house."

"You mean that it will not be free to members of the club who only come in sometimes?"

### Not a Public Room.

"That is so. My idea was that, however comfortable the public rooms might be, there would always be the chance of strangers dropping in, and thus those who lived in the house would be driven to their bedrooms if they wished for the privacy which is one of the charms of a sitting-room in a private house. Therefore, I have set apart one room for "family life" as it were, where those who desire can do needlework (there will be a big table for cutting out) or any occupation not suited for a general sitting-room."

"The extra room should be a great boon, I fancy. Will you tell me the tariff for meals?"

"Breakfast of tea, coffee, or cocoa, rolls, butter, and preserves, 6d.; with bacon, eggs, or fish, 9d.; lunch of two courses, 1s.; tea with cake, 6d.; dinner of three courses, 1s. 3d.; and supper of two courses, 1s."

"These, of course, are club prices. Have you any different charges for residents?"

"If they prefer they can be boarded for one and a-half guineas a week, or 5s. 6d. a day. These terms include the bedroom."

### The Lift.

"Now," continued Miss Debenham, "perhaps you would like to see over the house," and as we stepped into the lift she pointed out the way in which it is worked. It does not need an attendant; every member can use it when she chooses, and yet there are no risks. It is impossible to step into space, because the gates will not open until the lift is at rest opposite to them. Then the passenger steps in and presses a button to indicate at which floor she desires to alight, and the lift stops automatically. It is worked by electricity, and it seemed to me a wonderful invention.

### The Kitchens.

With the exception of the shops on the ground floor, the whole of St. Andrew's Home belongs to Miss Debenham, and we went to the top of the house first, where I admired the large, airy kitchens. Though the morning was a very warm one, I was struck with the fresh breeze which came in at the windows and with the cool atmosphere. The cooking is all done by gas, and the walls are entirely lined with brown and sea-green tiles. There is a lift to the dining-room. The entire fifth floor is devoted to domestic arrangements. There is a servants' hall, a good-sized bath-room, and ample bedroom accommodation for the eight female servants. The roof is flat, so that nurses desirous of a "blow" can easily obtain one, and the view on a fine day is extensive. I must not forget to mention the two iron outside staircases which reach down to the ground in case of fire, with a platform for entrance on each floor, so that those on the fifth storey are practically as safe from risk of burning as those on the first.

### Box and Bath Rooms.

Before we descended to the next floor I went into the box-room, where the nurses can store any surplus luggage they wish at a small charge, and around which are hanging cupboards, numbered and provided with locks, which can be rented by the inmates, temporary or permanent, of St. Andrew's Home. Hanging cupboards are also provided upon each floor, as well as two bath-rooms and lavatories. The bath-rooms are especially nice, lined with a special sort of tile called chrystopal, the predominant hues being pale green with a brown dado. On some of the floors there is also an ingenious contrivance so that the nurses can wash their hair when they wish. The water is conveyed up a pipe which projects and hangs directly over the basin, and the cold and hot supply is regulated by a screw.

### Room for Residents.

The third floor, Miss Debenham told me, she intends to be occupied entirely by residents, so as to again increase the feeling of home, and some of the rooms are very charming. Everywhere the colouring is most artistic, the green and terra-cotta, which is mostly used, toning in excellently together. Every room in the house has a fireplace, though the house itself is heated by a furnace in the basement, and the windows are large and light, many having delightful window seats. The bed-sitting-rooms, several of which were already furnished, look most inviting. The washstand is hidden by a screen, and the bed is specially designed so that it may, when needed, resemble a sofa. It is of green wood, the head and the foot being the same height, and during the day it is covered with a large terra-cotta rug, edged with sateen frills, a big pillow being made to match, so that the whole makes quite a pretty lounge.

THE DINING AND DRAWING ROOMS.

The public rooms are on the first floor, and are very handsome. The dining-room has two large fireplaces, with oak panelling and overmantels, the walls of a warm crimson hue, and round tables are employed. Attached is a small room which can be used for private luncheon or dinner parties. The drawing-room with a very long window seat in the great window, and a quaint little oriel recess in the corner is distempered in pale yellow, whilst the library is painted sealing-wax colour, and the walls covered with brown paper.

A SMOKING LOUNGE.

The spacious corridor is fitted up as a lounge, and here smoking is allowed, for the sake of male visitors, and also to prevent the reprehensible habit, against which Miss Debenham sets her face very strongly, of nurses smoking in their bed-rooms.

IN EVENT OF SICKNESS.

As I said good-bye to the founder of this really splendid club, I remarked how much I had been struck with the comfort of the arrangements. I said, "There is only one note of sadness, Miss Debenham. How hard it will be for the nurses to leave all this and go away if they fall ill." "But they need not," she replied. "I have especially set aside a completely isolated corner in case of sickness occurring, or a slight operation being necessary. There is an airy bedroom for the patient, a bed-room for the nurse adjoining, and a bath-room and lavatory to be used by no one else."

### Appointments.

ROYAL INFIRMARY, PRESTON.—Miss Beatrice A. Cookes has been appointed Assistant Matron. She was trained for three years at Leeds General Infirmary; subsequently she was night sister at the Metropolitan Hospital, Kingsland Road, London; day sister at the Manchester Children's Hospital, Pendlebury; and second assistant at the Nurses' Co-operation, 8, New Cavendish Street, W.

HORTON INFIRMARY, BANBURY.—Miss A. Wetherell has been appointed Matron. She was trained at St. Thomas's Hospital, and has since been staff nurse at St. Marylebone Infirmary, sister at the Royal Albert Edward Infirmary, and lately matron of the Albert Infirmary, Winsford, Cheshire.

GRANTHAM HOSPITAL.—Miss Emily J. Mildred has been appointed Matron. She was trained at the General Infirmary, Leeds.

### To Nurses.

WE invite contributions from any of our readers, and shall be glad to pay for "Notes on News from the Nursing World," or for articles describing nursing experiences, or dealing with any nursing question from an original point of view. The minimum payment for contributions is 5s., but we welcome interesting contributions of a column, or a page, in length. It may be added that notices of entertainments, presentations, and deaths are not paid for, but, of course, we are always glad to receive them. All rejected manuscripts are returned in due course, and all payments for manuscripts used are made as early as possible at the beginning of each quarter.

### The Nurses' Bookshelf.

[We invite Correspondence, Criticism, Enquiries, and Notes on Books likely to interest Women and Nurses. Address, Editor, THE HOSPITAL (Nurses' Book World), 28 & 29, Southampton Street, Strand, London, W.C.]

PRAYERS FOR USE IN HOSPITALS AND INFIRMARIES. (Society for Promoting Christian Knowledge, Northumberland Avenue, W.C. Pp. 31.)

THIS little collection of prayers and hymns is compiled with the distinct object of supplying what has been felt to be a need, and we are of opinion that it admirably fulfils its purpose. There are seven sets, or one set of prayers for each day in the week. They are very simple and short, merely consisting of the Lord's Prayer and responses, with suitable collects which brings them within the reach of all. The habit of commencing and ending the day with prayer is a practice which must commend itself to all who have the spiritual welfare of patients at heart, and in sickness is found just that opportunity of reaching the heart in an unobtrusive manner which this simple collection of prayers endeavours to supply. We have every confidence in recommending it to those who are in search of some such little work.

A HANDBOOK OF NURSING. By M. N. OXFORD. (London: Methuen and Co., Essex Street, W.C. Pp. 283. Price 3s. 6d.)

THERE are nowadays so many excellent handbooks on nursing that we should be tempted to describe the one before us as somewhat superfluous were it not for its intrinsic merits. Of course, much of it partakes of the nature of an oft-told tale, but it is put into fresh shape in an interesting and instructive fashion, which cannot fail to be attractive to the student of its pages. Each training school has its own distinctive teaching, and its customs differ in detail from those of its neighbours. For this reason alone it is well that Guy's Hospital should be represented in the world of nursing literature, and that its nurses should be equipped with a special textbook of their own. That Miss Oxford has acquitted herself worthily of the task was only to have been expected from a lady of her experience. The early chapters in the book bristle with useful hints, and the would-be probationer would do well to apply them and ask herself if she is prepared to carry out faithfully and intelligently the suggestions so clearly thrown out. The tendency of young nurses in the present day is to consider that they are either neglected or their powers underestimated if they are not always provided with acute cases, and continual excitement in the way of accidents, operations, &c., quite forgetting that the true nursing spirit is best manifested in the faithful care of chronic cases and others whose general condition of comfort alone speaks for the care that has been lavished so ungrudgingly. In Chapter X. there are some useful hints on medicine-giving and stimulants, and those chapters devoted to the application of remedies are among the most useful in the book. The different organs and their functions are separately described with the diseases peculiar to each, and their treatment from a nursing point of view; thus following on the description of the lungs we have the diseases of the respiratory tract. The remarks on diphtheria and the requirements for tracheotomy are admirable, and should be studied by all nurses. Some excellent sick-room recipes are contained in an appendix, and a vocabulary of medical terms is found in the concluding pages.

### Wants and Workers.

A NURSE-MATRON of a very isolated Cottage Hospital in a village 20 miles west of London, would be most grateful if some kind-hearted person would lend her books (not yellow-backs) gratis. No infectious cases. Great care taken of them. Address "Peaceful," c o J. Treland, St. Leonard's Road, Windsor.

### Echoes from the Outside World.

AN OPEN LETTER TO A HOSPITAL NURSE.

To say that sorrow and rejoicing go hand in hand is only to repeat a truism, but its veracity has been strangely apparent lately. On Saturday, amid mourning and lamentation, the Duke of Saxe-Coburg was laid to rest; on Sunday, with much ceremony, the King of Servia was united to Madame Draga Maschin; and on Thursday weeping Italy bids a last farewell to its murdered King. The account of the obsequies of "Our Sailor Prince" possesses, of course, special interest for us all. The first part of the service was held at noon, the time being fixed so as to allow of the German Emperor attending the ceremony. It took place in the church of St. Moritz, with the sunshine streaming through the windows and falling on the ivory crucifix at the head of the coffin and on the gleaming orders belonging to the late Duke which lay at the foot. But the interment itself followed nearly twelve hours later, when, with muffled drums, the hearse decorated with oak and pine, the soldiers bearing flaming torches of pine, and the moon looking quietly down upon the scene, the *cortège* made its way to the lovely cemetery upon the hills which was to be the last home of Prince Alfred. The wreaths sent by the Queen, with the simple words, "From his sorrowing mother," by the Prince of Wales and his family, and by the Princess herself, with the autograph inscription—"To dearest Alfred, in loving memory, from his devoted sister Alex. Sleep, our beloved, and take thy rest. Now comes peace. Good-night"—as well as those of his own family, were buried with him. It was evidently a trying day for the new little Sovereign, who is no longer called Duke of Albany, but Duke Charles Edward. When he drove to the station in the morning to meet the Kaiser, to quote from a Coburg paper, "The unanimous opinion was expressed that the Duke could not have conducted himself in a more pleasing, winning, and captivating manner than he had done on this, his first meeting with the Coburgers." Subsequently, when the Court Chaplain gave an address, and the boy's tears gathered fast, the Emperor laid a gentle hand on the boy's shoulder, and left it there a few moments as if to comfort him. At night it was the Prince of Wales, who, standing next to him during the last scenes, noticed the almost overwhelming fatigue of the young mourner, and, slipping his arm inside that of the boy, gave him the support of his kindly grasp. My sympathy is strong, too, for the Duchess of Albany, who must feel so anxious about her son, called, at least in theory, to rule so much sooner than was expected, at an age when most youths' highest ambition is a good score at cricket or a high place in class.

---

You may like to hear a little about the new King of Italy, Victor Emmanuel III. As a child he was weak and ailing, and manifested signs of rickets; but owing to the care of his English governess, who was particular that he should have plain fare, especially plenty of porridge, with fresh air in abundance, he became rapidly stronger. Unfortunately, as he grew older, his mother, who is herself extremely clever, was strongly imbued with the necessity of his becoming a good scholar, and had him kept closely to his books. He studied French, English, German, Latin, Greek, mathematics, philosophy, and military history till his health completely gave way, and his father insisted upon his being taken away from study and sent travelling for some time, which did much to restore him to strength again. He is said to be a perfect soldier, and is passionately fond of his profession, interesting himself in each man under him, quick to note incapacity, inexorable towards those who do not fulfil their duty, but ready enough to pardon slight errors. He married the beautiful Hélène, Princess of Montenegro.

Parliament was prorogued on Wednesday, and most people seem to think that it will not meet again. A dissolution in October is the general, but not the universal, expectation. Up to the very last the interest centred in China and South Africa. In respect to the former, the news continues to be conflicting, though a message from Sir Claude MacDonald leaves no room for doubt that the Legations were safe on the 3rd of August. Rumours, however, are current of renewed attacks since. The surrender of Harrismith to General MacDonald is the principal item of intelligence from South Africa, and there is fresh talk of the desire of President Kruger to surrender. But so long as he wants to dictate any of the terms Lord Roberts will, of course, refuse to listen to him. Still, I am glad to see that Mr. George Wyndham tacitly admits that he thinks the war may be over in "a few weeks."

---

As the Central London Electric Railway is now open to the public, I made a point of travelling along it one day last week, even though I lost a penny by doing so. My 'bus fare would have been only the humble copper, but on the Central Railway every ticket issued is the same price—2d.—which is very nice when you want to go a long way, but rather annoying when the journey is merely from one station to the next. A little point struck me at once. It is impossible to lose your ticket and have to pay over again, as often happens on an ordinary railway, for when you pass the barrier, instead of the ticket being "nipped," you are requested to throw it into a big receptacle as you go by—an official standing near to see that you do so. Next you find yourself awaiting the advent of the lifts, of which there are three or four, some with seats, others without. Each holds a hundred persons, and there have been complaints made because smoking is allowed. Considering how tightly the people are wedged in, the contention that the extra smell of tobacco, frequently bad or indifferent, is the last straw, seems to me perfectly legitimate. If a man objects to putting his pipe out he can always walk down the stairs. Those who are extremely bad sailors will probably adopt this course anyhow, after the first descent, for the oscillation is considerable, and the frequent jerks are strongly suggestive of a jibbing horse. As we emerged through the doorway at the bottom I had an experience for which I had not paid. I saw a sudden vision of clean, white-tiled passages, then blackness and darkness. The electric light had gone out. The porters took it so coolly that it was evidently not quite unexpected. They shouted out "Stand still!" once or twice; one or two women screamed faintly, many more gave vent to exclamations of amusement, and the light returned. I had heard this new railway laughingly called "The Refrigerator," and, indeed, it merits its name. The sudden transition from the atmosphere of the South Pole in the streets above to the atmosphere of the North Pole down below was most striking, and, had I been wearing a cotton or a muslin blouse, I should have been a little afraid of the consequences. It is true that the freezing process does not last long, as the train glides in at once, the trellised iron doors are opened by the guard inside, and you step on board. I travelled between three and four o'clock in the afternoon—a presumably slack time—but I found no sitting room. Every carriage was packed, and the leathern thongs hanging from the roofs to steady the upstanding passengers tell their own tale of anticipated pressure. At each stoppage the official calls out the name of the station and opens the gates for egress, closing them again as soon as the incoming passengers have passed through. As he does this he announces the name of the station which will come next, so that there shall be no excuse for being left behind, although the time of waiting at the platforms is strictly limited.

### Everybody's Opinion.

[Correspondence on all subjects is invited, but we cannot in any way be responsible for the opinions expressed by our correspondents. No communication can be entertained if the name and address of the correspondent is not given, as a guarantee of good faith but not necessarily for publication, or unless one side of the paper only is written on.]

### NURSING IN A FRONTIER HOSPITAL, BULAWAYO.

THE Chairman (Mr. E. Rose Townsend) of the Memorial Hospital, Bulawayo, Rhodesia, sends us a letter on the bearings of an article under the above heading which appeared in the "Mirror" on March 31st last, in the course of which he says: The article entitled "Nursing in a Frontier Hospital at Bulawayo" "by a Special Correspondent," and appearing in the "Nursing Mirror" of March 31st last, a supplement to your journal, has been brought to the notice of my board. As the article in question contains statements which are totally unfounded, and of a most damaging character against the management of the institution, I shall be glad if you will furnish me with the name and address of your correspondent, so that such steps may be taken as are deemed necessary. Readers of your journal who have friends in this country must naturally be alarmed at the prospect of their friends having to seek admission, in cases of illness, to an institution where, it is alleged, "lives are being thrown away through their (the nurses) carelessness and neglect." For your information I may state that the institution is governed by a board consisting of nine leading citizens, three of whom are permanently appointed by the Government and six nominated and appointed by subscribers to the institution, which is vested in trustees on behalf of the public of Rhodesia. The medical staff consists of two highly qualified and experienced English doctors, and an advisory board consisting of six of the leading private medical practitioners. The nursing staff consists of a matron, who is a British certificated nurse of wide experience in England and the Colonies; staff nurses who hold British or Colonial certificates, and who have been selected on account of their experience, and probationers who receive such training as to qualify them for obtaining University certificates. It is, therefore, ridiculous to suppose that such a state of affairs as is depicted in the article under consideration, and which is, on the face of it, an alleged contribution by a nurse who has admittedly only been engaged in the institution for a short period, could have escaped notice or be wilfully suppressed. My board is prepared to allow any public inquiry which may be deemed necessary to thoroughly investigate these charges against an institution which it has the privilege of administering, as it is quite confident that these charges have no foundation on fact. In view of the foregoing, my board trust and believe that you will take all steps which may be necessary to counteract so far as possible the harm which has been done by the article published by you, and which has been copied by other widely-circulated journals, and requests that in future no publication be allowed in your paper which may be detrimental to the interests of the Memorial Hospital until you have taken care to verify the correctness and *bona fides* of allegations against the institution.

⁂ The article in question refers to "a private hospital," and makes no mention of the Memorial Hospital, which, from the chairman's description, is a public institution. We are very glad to have the opportunity to publish the interesting particulars sent us by Mr. Townsend of the chief hospital in Bulawayo, which can in no way be affected by the article referred to.—ED. *T. H.*

### WORKHOUSE NURSING.

"PHYLLIS" writes: I attended a meeting of a board of guardians in June as one of three selected candidates. At our request the master came into the room where we waited to give particulars. The beds were 140, the patients there in June were 80—that would mean over 100 all the winter; the nurses five, including the superintendent. I knew the nursing could not be done well with the strength, so I asked if they allowed pauper nursing, to which the master replied that their cases required no nursing. "A few ulcerated legs, which might just as well dress themselves; about eight confinements per annum; and old women with rheumatism, who only asked a pipe to keep them happy. In fact a few pneumonias following influenza were all the cases requiring nursing which he had seen there; and as his wife was a nurse who had worked under the most eminent London surgeons they understood all that was required." I said, "What a pity you do not get your wife appointed superintendent nurse, with an assistant to take her stores work; it answers very well at S———, a much larger place." I also inquired after the M.O., and found that he lived in the town five miles away, and came out by train every day. When I got in the board room I wondered if I was asking for relief, so searching and minute was the questioning I had to undergo. What specially exercised the board was my having spent the winter at home, and my reason for leaving my last post, viz., that the north did not suit my chest. I was armed with a medical certificate of health and the testimonial of a clergyman residing near my home. That anyone should need a change from workhouse nursing to home life without being actually ill seemed beyond their belief. Their own leave was a fortnight per annum, which I told them was not enough, and that the staff was insufficient. One guardian inquired if I quite understood that it was a working nurse that was wanted. I replied that I expected to work, but I could not help adding, "The master says there is nothing to do." It was quite plainly intimated, though not in so many words, that no hospital ways were wanted there. I wish you would call attention to the fixed idea guardians seem to have that an "out of work" nurse is no good, so they appoint a nurse who has to go back and give a month's notice in preference to one who can come at once, thus depriving some other board of a member of their staff, encouraging nurses to change, and, worst of all, keeping their own infirmary some time without the necessary help.

### ASYLUM NURSING.

"AN ASYLUM HEAD NURSE" writes: Among the many employments for women nursing seems to be so popular that it is surprising to find one branch of that profession so often overlooked, viz., asylum nursing. This must be due to the fact that the general public have formed a very erroneous idea of what asylum work really is. A good-tempered, healthy, intelligent young woman can command a good salary from the very first, and if she puts her heart into her duties is certain of speedy promotion. Hospital nursing attracts many who have a sentimental notion of benefiting their fellow creatures, but the number of rejected applicants show that the supply is far greater than the demand, and if the enthusiastic girls who have to wait months, and even years, for admission to hospitals would turn their attention to asylums they would find unlimited opportunities of doing good to those poor creatures suffering from that worst of all maladies, insanity. The number of cases of nervous breakdown are becoming so numerous and require so much skilled attention that the question of providing nurses qualified to give such attention and to render useful service in the treatment of mental disease is a very important matter. The Medico-Psychological Association has done much in recent years to raise the standard of nursing in asylums by means of lectures on the theoretical, and instruction in the practical, part of the work. After two years' training examinations are held and certificates of proficiency given. This has helped to eliminate those who, through lack of intelligence or interest, have proved themselves unfit for the life, and has imbued the others with a deeper sense of the value and importance of their services.

### For Reading to the Sick.

"GIVING thanks always for all things unto God."—*Eph.* v. 20.

There are no disappointments to those whose wills are buried in the will of God.—*Faber.*

For blessings of the fruitful season,
For work, for rest, for friends and home,
For the great gifts of thought and reason,
To praise and bless Thee, Lord, we come.
Yes, and for weeping and for wailing,
For bitter hail and blighting frost,
For high hopes on the low earth trailing,
For sweet joys missed, for pure aims crossed.
—*E. Scudder.*

Notwithstanding all I have suffered, notwithstanding all the pain, the weariness, the anxiety and sorrow that necessarily enter into life, and the inward errings that are worse than all, I would end my record with a devout thanksgiving to the great Author of my being. For more and more am I unwilling to make my gratitude to Him what is commonly called "A thanksgiving for mercies" for any benefits and blessings that are peculiar to myself or my friends, or, indeed, to any man. Instead of this I would have it to be gratitude for all that belongs to my life and being—for joy and sorrow, for health and sickness, for success and disappointment, for virtue, for temptation, for life and death; because I believe that all is meant for good.—*Orville Dewey.*

I cannot say
Beneath the pressure of life's cares to-day
I joy in these;
But I can say
That I had rather walk this rugged way
If Him it please. —*S. G. Browning.*

We are ready to praise when all shines fair; but when life is overcast, when all things seem to be against us, when we are in fear for some cherished happiness, or in the depths of sorrow, or in the solitude of a life which has no visible support, or in a season of sickness, and with the shadow of death approaching—*then* to praise God; then to say, This fear, loneliness, affliction, pain, and trembling awe are as sure tokens of love, as life, health, joy, and the gifts of home: "The Lord gave, and the Lord hath taken away;" on either side it is He, and all is love alike; "blessed be the name of the Lord"—*this is the true sacrifice of praise.* What can come amiss to a soul which is so in accord with God? What can make so much as one jarring tone in all its harmony? In all the changes of this fitful life, it ever dwells in praise.—*H. E. Manning.*

In time of trouble go not out of yourself to seek for aid; for the whole benefit of trial consists in silence, patience, rest, and resignation. In this condition divine strength is found for the hard warfare, because God Himself fights for the soul.

### TRAVEL NOTES AND QUERIES.

RULES IN REGARD TO CORRESPONDENCE FOR THIS SECTION.—All questioners must use a pseudonym for publication, but the communication must also bear the writer's own name and address as well, which will be regarded as confidential. All such communications to be addressed "Travel Editor, 'Nursing Mirror,' 28, Southampton Street, Strand." No charge will be made for inserting and answering questions in the inquiry column, and all will be answered in rotation as space permits. If an answer by letter is required, a stamped and addressed envelope must be enclosed, together with 2s. 6d., which fee will be devoted to the objects of the "Hospital Convalescent Fund." Any inquiries reaching the office after Monday cannot be answered in "The Mirror" of the current week.

LAUSANNE (Inquirer).—Sorry for delay, but am on the Continent. Hotel Gibbon, opposite the post office; winter terms from 6 francs. Hotel Riche Mont has a nice garden; rather lower terms. Hotel du Nord, about the same terms. For pensions: Hotel National and Pension Gallo is in the Avenue de Beau Séjour, further in the town, near the Jura Simplon Station. Pension Campart, close to the English church; winter terms from 5 and 6 francs per day. It is not the custom to charge by the week on the Continent, but by the day, though the bills are settled weekly.

### Notes and Queries.

THE Editor is always willing to answer in this column, without any fee, all reasonable questions, as soon as possible.

But the following rules must be carefully observed:—

1. Every communication must be accompanied by the name and address of the writer.
2. The question must always bear upon nursing, directly or indirectly.

If an answer is required by letter a fee of half-a-crown must be enclosed with the note containing the inquiry.

*Pediculi.*

(181) A friend has a camp for poor little girls at the seaside, and is greatly distressed to find that the parasite Pediculus vestimentorum has gained ground. Kindly give me your advice as to the best manner of cleansing the camp.—*Anxiety.*

The only way to control these pediculi is by rigid insistence of cleanliness, and a liberal use of soap and water night and morning for all children in the camp. Carbolic lotion added to the water is a great assistance, and all clothing should be baked before going to the laundry. If the parasite has gained ground in the manner described, bedding as well as clothing may require to be baked. The children should certainly be washed all over every day with soft soap and water. If heads are affected, we find the best way of treating them is by combing night and morning with a small toothcomb and rubbing in carbolic lotion, one in 30 or 40, or methylated spirit, according to the case. If the head is very bad on admission a carbolised compress is applied and the hair cut short (or shaved) next day. With regard to the third form of pediculus, the same treatment as first described is usual and sufficient. Sometimes a sulphur bath or ointment is ordered, but only in special cases.

*Rome or Nice.*

(182) "M." writes: Referring to Answer 168 for July 28th, Miss Martin has left St. Paul's Home in Rome, and her place was taken by Miss Watson, who has removed to 18, Piazza Barberini. The Home of the Little Company of Mary employ only English nuns, not lay nurses.

*Nurses' Reading Society.*

(183) Can you tell me the address of the Secretary of the above society?—*Policy No. 7,615, and S.E.S.*

Miss L. G. Moberly, 24, Portland Place, W.

*Midwifery.*

(184) Would you kindly let me know where I could be trained in midwifery for the sum of £7?—*K. A.*

Write to the Secretary, the Midwives' Institute, 12, Buckingham Street, Strand.

*Colonial Nursing Association.*

(185) Will you kindly tell me the address of the "Colonial Nursing Institute"?—*Nurse Annie.*

The Imperial Institute, S.W.

*Monthly Nursing.*

(186) Will you kindly tell me which would be the best hospital at which to learn monthly nursing? Also, what would the fee be for three months' instruction?—*A Reader.*

There are a number of good hospitals, the cheapest being the City of London Lying-in Hospital, City Road, fees £7 7s.; the British Lying-in Hospital, Endell Street, fees £7 3s.; and the East-End Mothers' Home, Commercial Road, E.

*Passage with Patient.*

(187) Can you tell me where a nurse could hear of a patient going to Chicago who would pay her passage in return for services? She wishes to join her sister, who is a nurse there.—*Nora W.*

You can only advertise and watch advertisements in the usual way.

*Bed Support.*

(188) Can you inform me where I can procure the "Coventry Bed Support?"—*Sister Barbara.*

You probably mean the "Excelsior Bed Support," supplied by the Bed Support Company, The Baths, Coventry.

*Pharmacopœia.*

(189) 1. Can you tell me a cure for chronic hay fever? 2. Can you give me the price of "British Pharmacopœia," also if there is a similar book published, only cheaper?—*Nurse.*

1. No; we cannot prescribe for our correspondents. 2. 10s. 6d. There are many small books on the subject.

*Holiday Homes.*

(190) Will you kindly inform me whether there are any holiday homes for nurses at Yarmouth or Lowestoft?—*M. M.*

If you write to the Matron, Great Yarmouth Hospital, or to the Matron, Lowestoft Hospital, they will probably be able to help you.

### ANSWERS REQUESTED.

*Photograph of Miss Nightingale.*

(191) Will you kindly let me know where I could get a photograph or engraving of Florence Nightingale?—*Watchful.*

**Standard Books of Reference.**

"The Nursing Profession: How and Where to Train." 2s. net; post free, 2s. 4d.

"The Nurses' Dictionary of Medical Terms." 2s.

"Burdett's Series of Nursing Text-Books." 1s. each.

"A Handbook for Nurses." (Illustrated.) 5s.

"Nursing: Its Theory and Practice." New Edition. 3s. 6d.

"Helps in Sickness and to Health." Fifteenth Thousand. 5s.

All these are published by THE SCIENTIFIC PRESS, Ltd., and may be obtained through any bookseller or direct from the publishers, 28 & 29, Southampton Street, London, W.C.

